# CT-integrated pathophysiology-guided, clinically causal cognition-led interpretable lesion context characterization and risk reasoning in lung MWA

**DOI:** 10.3389/fmed.2025.1682823

**Published:** 2025-12-04

**Authors:** Meng Li, Yongzhao Li, Xiangming Wang, Hui Feng, Yang Li, Xinbo Liu

**Affiliations:** 1 Department of Radiology, The Fourth Hospital of Hebei Medical University, Shijiazhuang, Hebei, China; 2 Center of Treatment of Myasthenia Gravis, People’s Hospital of Shijiazhuang Affiliated to Hebei Medical University, Shijiazhuang, Hebei, China; 3 Department of Thoracic Surgery, The Fourth Hospital of Hebei Medical University, Shijiazhuang, Hebei, China

**Keywords:** non-small cell lung cancer, microwave ablation, interpretable diagnostics, clinical causal cognition, computed tomography

## Abstract

**Background:**

Accurate preoperative prediction of complication risks in microwave ablation (MWA) for non-small cell lung cancer (NSCLC) is critical yet challenging due to the complex anatomical context. Beyond feature heatmaps, clinicians need decision rationales that align with pathophysiology. We therefore frame the task as an interpretable lesion-context characterization driven by clinical causal cognition, where imaging-derived concepts (e.g., tumor–pleura distance, vascular proximity, pleural adhesions) are organized into traceable causal pathways supporting downstream risk reasoning.

**Objective:**

This study aimed to validate that a previously developed multi-task, attention-enhanced framework can be re-expressed through a concept bottleneck and lesion-context graph to provide pathophysiology-consistent, interpretable reasoning for lesion characterization and to support downstream prediction of pneumothorax, hemorrhage, and pleural reactions.

**Methods:**

This study retrospectively analyzed 184 NSCLC-MWA cases with paired thin-slice CT and expert annotations. Our architecture integrates a multi-scale cross-spatial attention-enhanced 3D U-Net and multi-task heads. On top of the existing pipeline, we introduce a concept bottleneck layer (tumor volume/shape, minimum tumor–pleura distance, vascular proximity, pleural adhesions), a post-hoc lesion-context graph with message passing for explanation, and a causal-consistency evaluation suite (directionality/monotonicity/sign tests, cross-subgroup invariance, counterfactual sensitivity). Human–AI collaboration was assessed by radiologists’ usability scores for the generated causal pathways. No retraining was required.

**Results:**

The framework maintained anatomical segmentation performance (Dice: tumor 0.878, vessels 0.851, adhesions 0.863) and multi-task AUCs (0.903/0.871/0.847 for pneumothorax/hemorrhage/pleural reactions). Causal-consistency showed expected negative monotonicity between tumor-pleura distance and pneumothorax risk (Kendall’s τ=+ −0.61; violation rate 9%), positive between vascular proximity and hemorrhage ( τ=+ 0.57; violations 12%), and positive between adhesions and pleural reactions ( τ=+ 0.49; violations 16%). Counterfactual geometric edits (±5 mm pleural distance; ±3 mm vascular proximity) produced direction-consistent risk changes in 86 and 83% of cases, respectively.

**Conclusion:**

By translating imaging features into clinically meaningful, traceable causal frameworks, our hybrid system aligns AI logic with clinical cognition for interpretable lesion-context diagnosis and reliable downstream risk reasoning. This “from black-box to clarity” shift improves trustworthiness and potential clinical utility.

## Introduction

Non-small cell lung cancer (NSCLC) remains the leading cause of cancer-related mortality worldwide, comprising approximately 85% of all lung cancer cases (1). For early-stage NSCLC, surgical resection remains the gold standard; however, many patients are deemed medically inoperable due to comorbidities or poor pulmonary reserve (2). In this scenario, minimally invasive interventions such as microwave ablation (MWA) have emerged as viable alternatives, offering effective local tumor control and reduced recovery times (3, 4).

Nonetheless, MWA is associated with several risks, including pneumothorax, hemorrhage, and pleural reactions. A retrospective study of 183 patients undergoing MWA for lung tumors reported pneumothorax as the most common complication (28.8%), followed by pleural effusion (24.6%) and pneumonia (8.2%), with major complications occurring in 3.8% of cases (5). Traditional risk assessments, which rely on clinical experience and basic imaging features (e.g., tumor size, distance to pleura), may not fully capture the complex spatial relationships between tumor location, surrounding anatomical structures, and complication risks (6, 7). Clinicians also require lesion-centered reasoning that clearly links anatomical context (tumor-pleura-vessels- adhesions) to procedural risks in a way that reflects clinical thinking rather than opaque correlations.

In recent years, deep learning (DL), particularly convolutional neural networks (CNNs), has shown remarkable advances in medical image analysis, enabling accurate volumetric segmentation and classification (8, 9). The 3D U-Net model effectively captures spatial contextual information for anatomical segmentation tasks (10). Additionally, spatial attention mechanisms have been shown to enhance model performance by enabling networks to focus on the most relevant regions of medical images, improving both accuracy and interpretability (11). Yet, attention maps alone may not constitute clinically acceptable explanations, as they lack direct pathophysiological meaning needed for decision support.

Simultaneously, multi-task learning methods—where the model jointly predicts related outcomes—have demonstrated enhanced performance in medical imaging by leveraging the shared representations across tasks, leading to improved generalization and computational efficiency (12). Despite these advances, few studies to date have integrated spatial attention within a multi-task framework to predict multiple correlated complications in NSCLC-MWA.

To close the gap between high predictive performance and clinical interpretability, we reformulated our previous multi-task attention framework as a system that follows clinicians’ causal reasoning about lesions and their context. First, we extract a small set of clinically meaningful concepts (tumor volume and shape, tumor-pleura distance, vascular proximity and pleural adhesions). Second, we build a post-hoc lesion-context graph to display possible causal pathways between these concepts and complications. Third, we test whether the model’s behavior is consistent with expected clinical directions across patient subgroups and under counterfactual edits. Finally, we assess how radiologists use and trust these explanations in simulated decision making. Complication risk prediction is thus supported by this interpretable diagnostic layer.

## Materials and methods

### Patient cohort preparation

**Figure 1:**
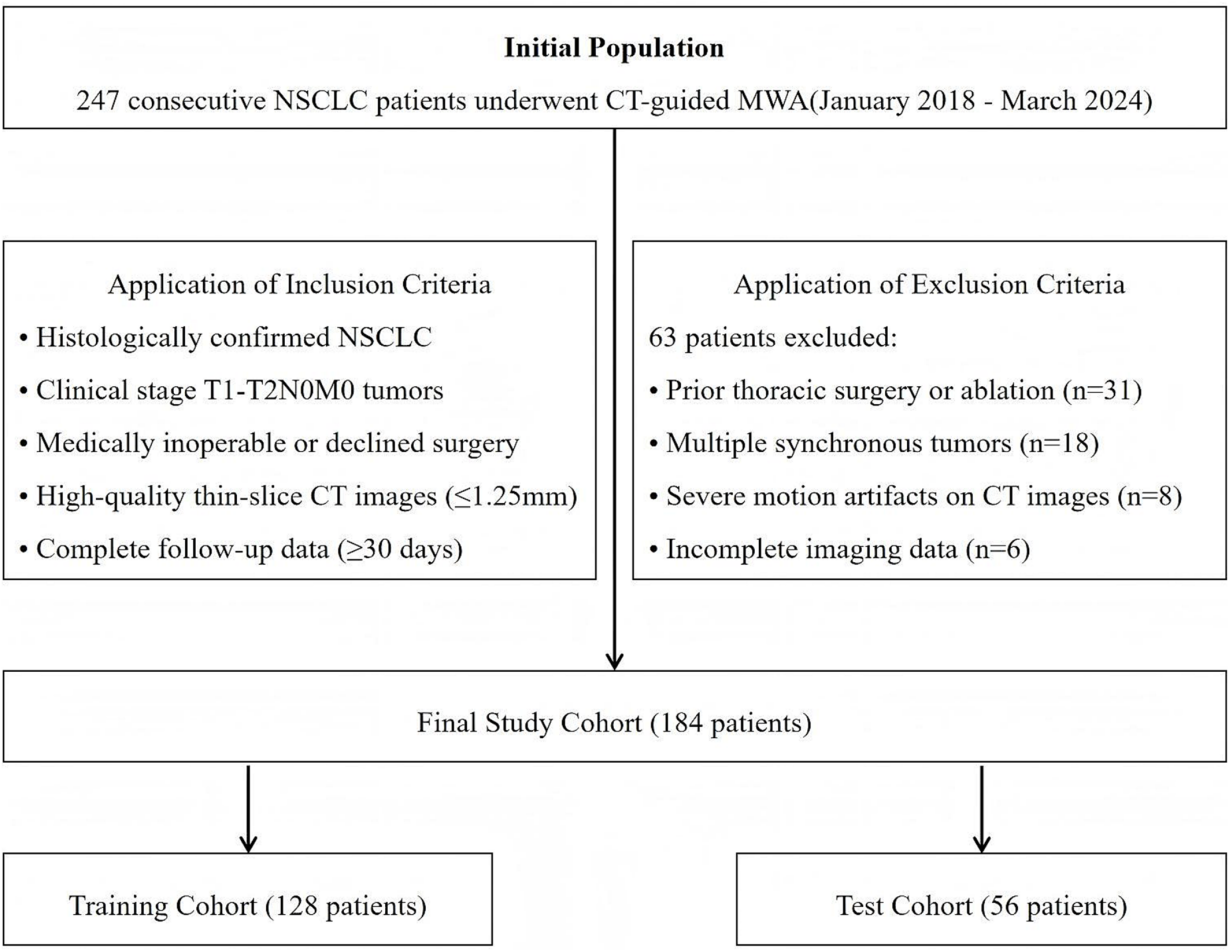
Flow diagram of the study population.

This retrospective study was conducted at our institution following approval by the Institutional Review Board (2025KT165). Written informed consent was waived due to the retrospective nature of the study. We identified 247 consecutive NSCLC patients who underwent CT-guided MWA between January 2018 and March 2024. Inclusion criteria were: (1) histologically confirmed NSCLC; (2) clinical stage T1-T2N0M0 tumors; (3) medically inoperable or patients who declined surgery; (4) availability of high-quality pre- and post-procedural thin-slice CT images (≤1.25 mm slice thickness); and (5) complete clinical follow-up data for at least 30 days post-procedure. Exclusion criteria included: (1) prior thoracic surgery or ablation (*n* = 31); (2) multiple synchronous tumors (*n* = 18); (3) severe motion artifacts on CT images (*n* = 8); and (4) incomplete imaging data (*n* = 6). The final cohort comprised 184 patients with a mean age of 68.4 ± 9.7 years (range: 45–82 years), including 108 males (58.7%) and 76 females (41.3%) (Figure 1).

### Image acquisition and preprocessing

All CT examinations were performed using a GE Revolution CT scanner (General Electric, United States), with standardized protocols. This model is part of GE’s ultra-high-end Revolution series and is equipped with a 256-row (160-mm z-coverage) Gemstone Clarity Detector, ultra-fast 0.28-s gantry rotation speed, and high-definition spatial resolution (<0.30 mm, up to 0.23 mm). The system supports Gemstone Spectral Imaging (GSI) via ultrafast 0.25-ms kVp switching, enabling collection of spectral CT data as described in the Discussion section.

Pre-procedural contrast- enhanced CT was acquired with the following parameters: tube voltage 100 kVp, automatic tube current modulation (reference 150 mAs), slice thickness 1.0 mm, reconstruction interval 0.8 mm, matrix 512 × 512, and field of view 350–400 mm. Post-procedural non-contrast CT was obtained immediately after MWA and at 24–48 h to assess for early complications. In addition, for patients who were clinically suspected of delayed inflammatory responses (e.g., new chest discomfort, low-grade fever, or elevated inflammatory markers), an additional follow-up CT was performed between 48 and 72 h as part of routine clinical care. This extended window enabled accurate identification of delayed-onset pleural reactions.

Furthermore, a routine follow-up CT examination was performed approximately 1 month after the procedure to evaluate ablation zone evolution and confirm complete tumor ablation, in accordance with institutional clinical practice guidelines.

Image preprocessing involved multiple standardized steps. First, Digital Imaging and Communications in Medicine (DICOM) files were converted to NIfTI format and resampled to isotropic voxel spacing of 1.0 × 1.0 × 1.0 mm^3^ using trilinear interpolation. Hounsfield unit (HU) values were normalized to the range [−1,000, 400] and subsequently rescaled to [0, 1]. To enhance model robustness, we applied data augmentation techniques including random rotation (±15°), elastic deformation (alpha = 100, sigma = 10), Gaussian noise addition (mean = 0, std. = 0.05), and intensity scaling (factor range: 0.9–1.1). The lung region was automatically extracted using intensity-based thresholding (HU < −500) followed by morphological operations to remove airways and vessels.

### Ground truth annotation

Ground truth annotations were performed by two experienced thoracic radiologists (with 8 and 12 years of experience, respectively) using Darwin AI Research Platform. The annotation protocol included precise delineation of: (1) primary tumor boundaries; (2) major pulmonary vessels (diameter ≥2 mm) within a 3 cm radius of the tumor; (3) pleural surfaces and adhesions; and (4) anatomical risk zones defined as regions with high complication probability based on spatial proximity to critical structures.

Complication outcomes were defined as follows: pneumothorax was classified as any detectable air in the pleural space on post-procedural CT; hemorrhage included both intrapulmonary bleeding (≥2 cm hematoma) and hemoptysis requiring intervention; pleural reactions encompassed pleural effusion, pleural thickening, or pleuritis. Inter-observer agreement was assessed using the Dice similarity coefficient for segmentation tasks (mean Dice: 0.91 ± 0.04) and Cohen’s kappa for complication classification (*κ* = 0.89, indicating excellent agreement). Discrepancies were resolved through consensus reading by a third senior radiologist.

For post-hoc interpretability analyses, we derived a set of lesion-context concepts from the expert-verified segmentations, including tumor volume and shape descriptors, the minimum tumor–pleura distance (mm) and pleural contact area (mm^2^), vascular proximity (minimum Euclidean distance to vessels ≥2 mm along with nearest caliber), and pleural adhesion presence/extent.

These derived descriptors were subsequently used in downstream interpretability and causal-consistency analyses.

### Multi-scale cross-spatial attention-enhanced 3D U-net

**Figure 2:**
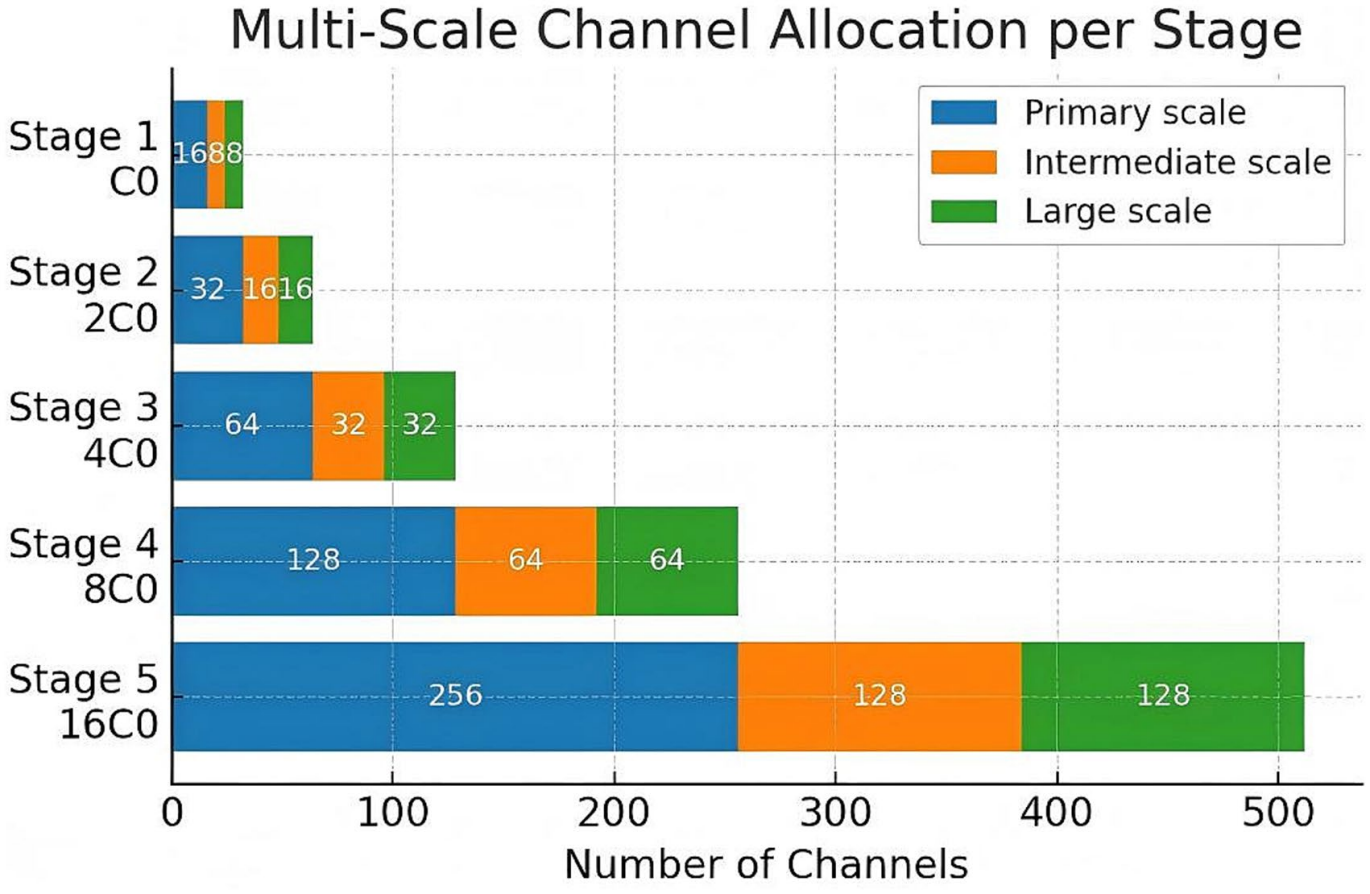
Channel allocation diagram.

The core segmentation network was based on a modified 3D U-Net architecture integrated with an Efficient Multi-Scale Attention Module with Cros82s-Spatial Learning (EMSA-CSL) to capture long-range dependencies and multi-scale contextual features with reduced computational overhead. The encoder comprised five resolution levels, where the base channel width $C_{0}$ was progressively expanded across stages $\left\{C_{0},2C_{0},4C_{0},8C_{0},16C_{0}\right\}$ (Figure 2). In our configuration, $C_{0}$ was set to 32, but each EMSA-CSL block internally reallocated the stage channels into multiple scale-specific sub-branches (e.g., 50% for the primary scale, 25% for intermediate scale, and 25% for the largest receptive field) to achieve efficient multi-scale feature learning with minimal parameter overhead.

At each encoder level, the EMSA-CSL module was inserted after the convolutional block. The EMSA-CSL design decomposes spatial attention into parallel sub-branches operating at different scales to efficiently aggregate local and global contextual information. Cross-spatial learning is achieved by fusing inter-branch features via element-wise addition and channel reweighting, formulated as:


$$Y=\sigma (\sum_{s\in S}W_{s}\cdot F_{s}(X))⊙X$$

where X denotes the input feature maps, S represents the set of scale-specific branches, $F_{s}$ denotes the convolution operation at scale s , $W_{s}$ are learnable channel weights, σ(·) is the sigmoid function, and ⊙ indicates element-wise multiplication. This formulation enables multi-scale feature recalibration without introducing excessive parameters or FLOPs.

**Figure 3:**
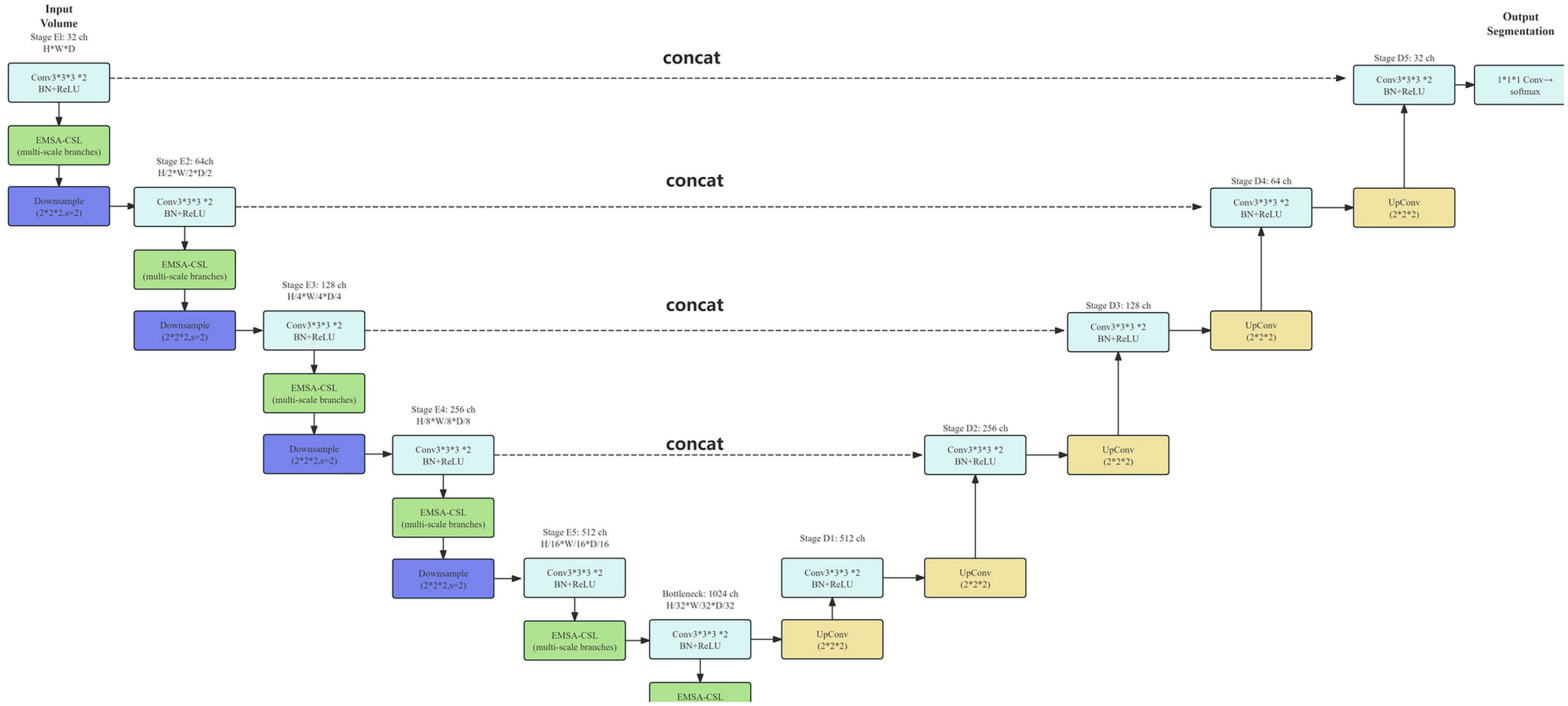
3D U-Net + EMSA-CSL model architecture diagram.

Additionally, a distance-aware gating mechanism was incorporated to refine object boundaries:


G=α·tanh(β·SDF)+γ

where SDF represents the signed distance function computed from initial segmentation predictions, and α,β,γ are learnable parameters. The gated output modulates the decoder features, thereby improving delineation of fine anatomical structures (Figure 3).

### Multi-task complication prediction module

The multi-task learning architecture included specialized branches tailored to accurately predict specific complication types associated with microwave ablation.

The Pneumothorax Prediction Branch was designed to capture multi-scale contextual information pertinent to pleural-lung interfaces. This branch incorporated Atrous Spatial Pyramid Pooling (ASPP) with dilation rates of Siegel et al. (1), Ye et al. (6), Zhang and Yang (12), and Dai et al. (13), enabling the effective detection of spatial features at various scales. It consisted of parallel atrous convolutional layers utilizing 3 × 3 × 3 convolution kernels to capture distinct receptive fields and anatomical contexts. A global average pooling operation provided comprehensive contextual embedding of spatial features, followed by the concatenation of multi-scale feature maps and a subsequent 1 × 1 × 1 convolutional layer for efficient dimensionality reduction and feature integration. Fully connected layers [512, 256, 128, 1], accompanied by dropout regularization (probability *p* = 0.3), were included to enhance generalization and mitigate overfitting.

The Hemorrhage Prediction Branch employed deformable convolutions to model irregular shapes and complex vessel geometries, focusing specifically on vessel tortuosity and potential bleeding sites. The deformable convolution operation is mathematically formulated as:


$${\text{Deformable}}_{\text{Conv}}(p)=\sum_{n}w(p_{n})\times x(p+p_{n}+\Delta p_{n})$$

where $p_{n}$ represents learnable spatial offsets dynamically adapting to geometrical variations inherent in pulmonary vasculature. This adaptive capability facilitated precise identification of hemorrhage-prone regions, thereby improving prediction accuracy.

The Pleural Reaction Branch incorporated texture-sensitive convolutions utilizing multi-orientation Gabor filters to detect subtle pleural changes indicative of inflammatory reactions or pleural thickening post-ablation. This branch employed Gabor filtering across eight orientations and three scales to robustly characterize complex pleural textures. Texture features were extracted through local binary pattern (LBP) analysis, effectively capturing subtle intensity variations and local textural details. An attention-weighted feature aggregation mechanism was implemented to emphasize diagnostically significant texture features, enhancing the sensitivity and specificity of pleural reaction predictions.

**Figure 4:**
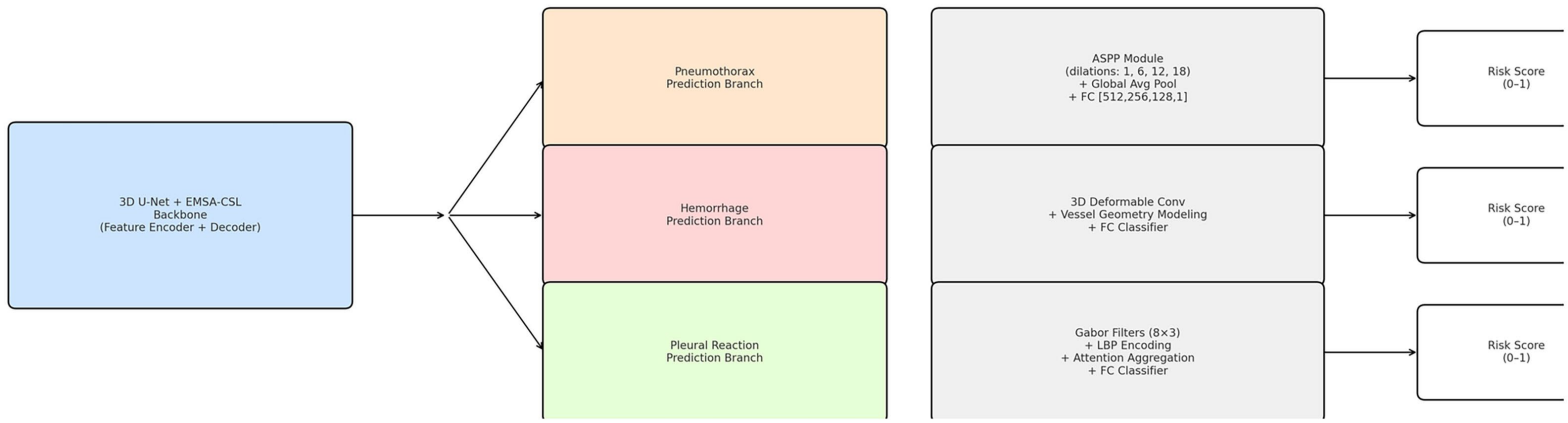
Overall network model architecture diagram.

**Figure 5:**
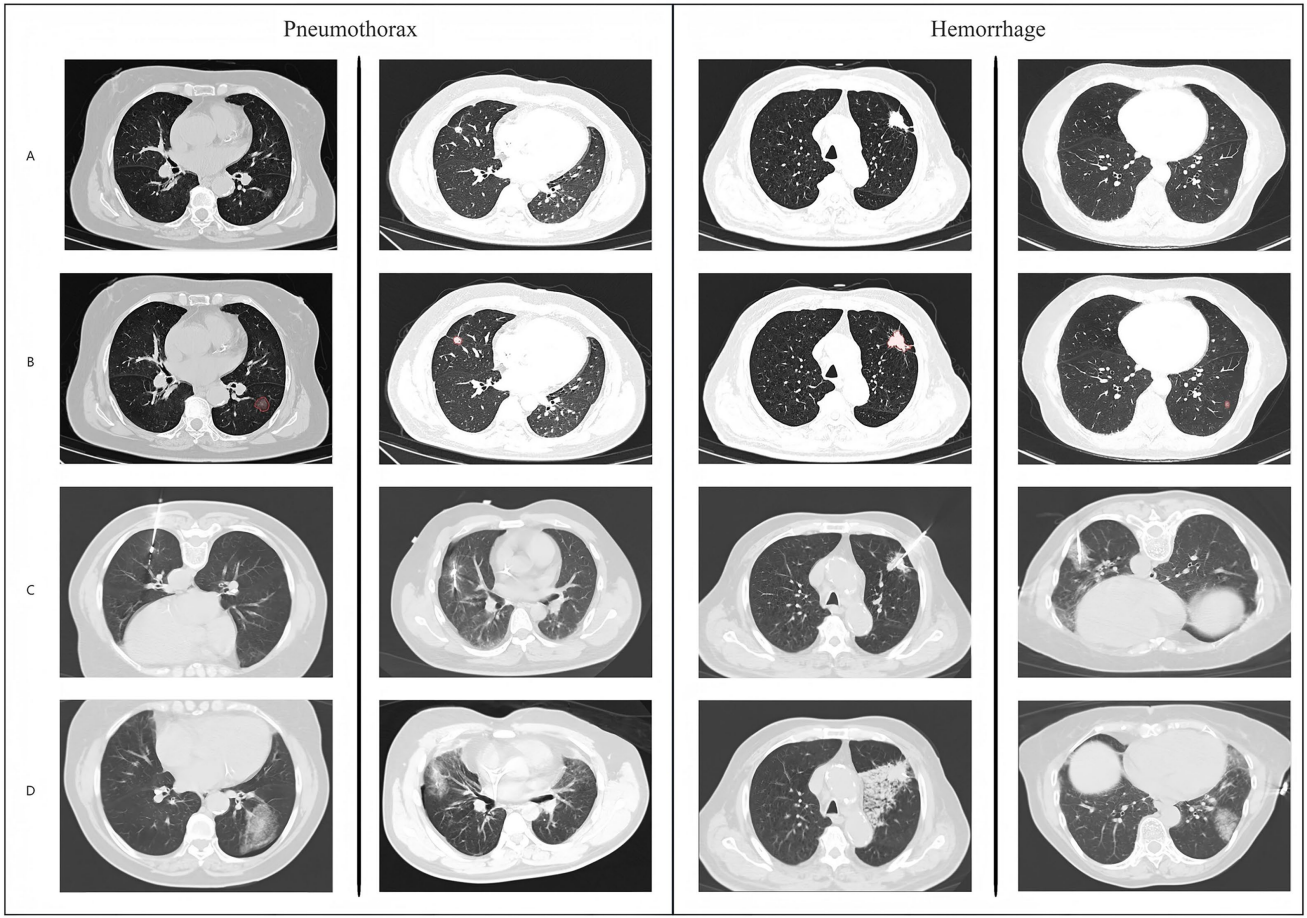
Schematic diagram and example data of model operation results.

These specialized prediction branches were integrated within the multi-task framework to maximize predictive performance and clinical applicability, effectively modeling distinct yet interrelated complication risks (Figures 4, 5). while maintaining shared feature representations across tasks.

### Loss function and training strategy

A composite loss function combining segmentation, classification, and regulariza -tion objectives was formulated to effectively optimize the multi-task model:


$$L_{\text{total}}=\lambda_{\mathrm{seg}}\times L_{\mathrm{seg}}+\lambda_{\mathrm{cls}}\times L_{\mathrm{cls}}+\lambda_{\mathrm{reg}}\times L_{\mathrm{reg}}$$

The segmentation loss $L_{\mathrm{seg}}$ integrated Dice loss and focal loss to robustly handle class imbalance and enhance the segmentation performance:


$$L_{\mathrm{seg}}=1-\text{Dice}+\alpha \times L_{\text{Focal}}$$

where:


$$\text{Dice}=\frac{2\times |P\cap G|}{|P|+|G|},\;\;L_{\text{Focal}}=-\alpha {\left(1-p_{t}\right)}^{\gamma }\log(p_{t})$$

Here, P and G represent the predicted and ground truth masks, respectively, and hyperparameters were set as (= 0.25) and (= 2.0).

For the classification tasks, weighted binary cross-entropy with class balancing was applied to effectively address class imbalances:


$$L_{\mathrm{cls}}=-\sum_{i}w_{i}\times [y_{i}\log(\hat{y_{i}})+(1-y_{i})\log(1-\hat{y_{i}})]$$

Class-specific weights $w_{i}$ were inversely proportional to class frequencies, set as $w_{\text{pneumot}h\text{orax}}=1.8,w_{h\text{emorr}h\mathrm{age}}=2.3,\text{and}\;w_{\text{pleura}l_{r}\text{eaction}}=1.6$ .

The regularization term $L_{\mathrm{reg}}$ incorporated L2 weight penalty $\left(\lambda_{\mathrm{reg}}=1\times 10^{-4}\right)$ and consistency loss between multi-scale predictions to further enhance model stability and generalization. Based on validation performance, hyperparameters were set as $\lambda_{\mathrm{seg}}=1.0,\lambda_{\mathrm{cls}}=0.5,\text{and}\;\lambda_{\mathrm{reg}}=0.1$ .

### Model training and validation

The dataset was partitioned using stratified random sampling to ensure representative distribution across training (*n* = 128, 69.6%) and testing sets (*n* = 56, 30.4%). Stratification was performed based on complication rates for each outcome variable (pneumothorax, hemorrhage, pleural reactions) to maintain balanced class distributions across all subsets. Additionally, stratification considered tumor characteristics (size, location, histological subtype) and patient demographics to minimize selection bias and enhance external validity.

The multi-task deep learning framework was implemented using PyTorch 1.12.0 with TorchVision 0.13.0 for advanced data augmentation pipelines. Model training and evaluation were conducted on a high-performance computing cluster equipped with NVIDIA Tesla V100 GPUs (32GB VRAM each) utilizing CUDA 11.6 and cuDNN 8.4.0 for optimized deep learning operations. The computational environment included Ubuntu 20.04 LTS, Python 3.8.10.

Model optimization employed the AdamW optimizer with an initial learning rate of 1 × 10^−3^, *β*₁ = 0.9, β₂ = 0.999, and weight decay *λ* = 1 × 10^−4^ to prevent overfitting. A cosine annealing learning rate scheduler was implemented over 200 training epochs, providing smooth learning rate decay according to: LR(t) = LR_min + (LR_max−LR_min) × (1 + cos(πt/T_max))/2, where t represents the current epoch and T_max denotes the maximum epoch count. Batch size was constrained to 2 due to GPU memory limitations when processing high-resolution 3D volumes (typical dimensions: 256 × 256 × 128 voxels). To mitigate potential gradient instability from small batch sizes, gradient accumulation across 4 mini-batches was employed, effectively simulating a batch size of 8.

### Baseline comparison models

Comprehensive performance evaluation was conducted against multiple established methodologies representing current clinical practice and state-of-the-art computational approaches. A conventional logistic regression model incorporating well-established clinical risk factors was developed as the primary clinical baseline. Input variables included quantitative imaging metrics (tumor diameter, minimum distance to pleural surface, tumor volume), patient demographics (age, gender, body mass index), clinical parameters (forced expiratory volume in 1 s [FEV₁], smoking history pack-years), and procedural factors (approach angle, number of electrode insertions). Feature selection utilized backward stepwise regression with Akaike Information Criterion optimization to identify the most predictive clinical variables.

A standard 3D ResNet-50 adapted for medical imaging was implemented for individual complication prediction tasks without multi-task learning capabilities, serving as a benchmark for evaluating the benefits of multi-task learning and spatial attention mechanisms. Additionally, a vanilla 3D U-Net architecture without spatial attention mechanisms was employed for anatomical segmentation tasks to isolate the contribution of attention modules to overall performance improvement.

A comprehensive radiomics pipeline was developed incorporating traditional handcrafted feature extraction methodologies. A total of 485 quantitative imaging features were systematically extracted from regions of interest, encompassing morphological features (*n* = 18) including shape-based descriptors such as surface area, sphericity, compactness, and maximum 3D diameter; first-order statistical features (*n* = 126) characterizing intensity distribution including mean, variance, skewness, kurtosis, and percentile-based metrics; and second-order textural features (*n* = 341) derived from Gray Level Co-occurrence Matrix (GLCM), Gray Level Run Length Matrix (GLRLM), Gray Level Size Zone Matrix (GLSZM), and Gray Level Dependence Matrix (GLDM). Feature selection employed minimum redundancy maximum relevance (mRMR) algorithm followed by recursive feature elimination to identify the most discriminative subset. Classification utilized ensemble random forest with 500 decision trees and hyperparameter optimization via grid search cross-validation.

Individual specialized networks were trained separately for each complication type to evaluate the advantages of multi-task learning architecture. Each single-task model employed identical network architectures to their corresponding branches in the multi-task framework but was trained independently without shared feature representations or joint optimization objectives. Model performance comparison utilized multiple evaluation metrics including area under the receiver operating characteristic curve (AUC-ROC), sensitivity, specificity, positive predictive value, negative predictive value, and F1-score. Statistical significance was assessed using DeLong’s test for AUC comparisons and McNemar’s test for paired binary classification outcomes, with Bonferroni correction applied for multiple comparisons (*α* = 0.01).

### Post-hoc interpretable lesion-context and clinical causal cognition analyses

Using the fixed model outputs and exper-derived masks, we calculated a set of per-case lesion-context concepts, including tumor volume, surface area, elongation and sphericity; minimum tumor-pleura distance and pleural contact area; the distance and caliber of the nearest vessel; and the extent of pleural adhesions. All measurements were obtained on resampled 1 mm isotropic CT volumes, and concept values were z-scored in the training set and then applied to the test set to keep reporting consistent.

We constructed a post-hoc lesion-context graph with nodes {tumor, pleural regions, adhesion patches, vessel segments} and edges encoding spatial relations (distance/contact/crossing). One-to-two rounds of lightweight message passing were applied solely to summarize contextual interactions into human-readable pathway panels (e.g., “shorter tumor–pleura distance → higher pneumothorax risk”). This step did not feed back into training or alter predictions.

We assessed whether concept–risk relations matched clinical expectations using: (i) directionality/monotonicity/sign tests (Kendall’s *τ*; isotonic regression violation rate); (ii) cross-subgroup invariance across age (<70/≥70), sex, COPD, tumor size (<2/2–3/>3 cm), lobe, and vendor/scanner strata (slope/direction stability); and (iii) counterfactual sensitivity via controlled geometric edits on fixed masks—expanding the tumor–pleura distance by +5 mm (3D morphological operations along surface normals), reducing vascular proximity by −3 mm, or toggling adhesion masks—and re-running the frozen network to verify direction-consistent risk changes.

Two to three board-certified thoracic radiologists (≥8 years) independently rated the clinical usability and trust of per-case pathway panels (graph + key concepts + predicted risks) on a 5-point Likert scale and indicated whether management would change; inter-rater reliability was quantified by ICC (two-way random). All assessments were performed on de-identified test cases under the same IRB approval.

## Results

### Patient demographics and clinical characteristics

The final cohort comprised 184 patients with histologically confirmed NSCLC who underwent CT-guided microwave ablation. The mean patient age was 68.4 ± 9.7 years (range: 45–82 years), with a male predominance of 108 patients (58.7%) and 76 females (41.3%). Adenocarcinoma was the most prevalent histological subtype (*n* = 132, 71.7%), followed by squamous cell carcinoma (*n* = 41, 22.3%) and other NSCLC subtypes (*n* = 11, 6.0%). The majority of patients presented with clinical stage T1N0M0 disease (*n* = 147, 79.9%), while 37 patients (20.1%) had T2N0M0 tumors. Mean tumor diameter was 2.8 ± 1.2 cm (range: 1.1–4.8 cm), with a median distance to pleural surface of 1.4 cm (Table 1).

**Table 1:** Patient demographics and clinical characteristics.

Characteristic	Value
Demographics	
Total patients, *n*	184
Age, years, mean ± SD (range)	68.4 ± 9.7 (45–82)
Male sex, *n* (%)	108 (58.7)
Female sex, *n* (%)	76 (41.3)
Histological subtype	
Adenocarcinoma, *n* (%)	132 (71.7)
Squamous cell carcinoma, *n* (%)	41 (22.3)
Other NSCLC subtypes, *n* (%)	11 (6.0)
Clinical stage	
T1N0M0, *n* (%)	147 (79.9)
T2N0M0, *n* (%)	37 (20.1)
Tumor characteristics	
Tumor diameter, cm, mean ± SD (range)	2.8 ± 1.2 (1.1–4.8)
Distance to pleural surface, cm, median (IQR)	1.4 (0.8–2.6)
Pulmonary function	
FEV₁, L, mean ± SD	1.9 ± 0.6
FEV₁, % predicted, mean ± SD	68.4 ± 18.7
Smoking history	
Current/former smokers, *n* (%)	156 (84.8)
Pack-years, mean ± SD	42.6 ± 23.1
Never smokers, *n* (%)	28 (15.2)
Comorbidities	
COPD, *n* (%)	89 (48.4)
Cardiovascular disease, *n* (%)	76 (41.3)
Diabetes mellitus, *n* (%)	34 (18.5)
Reason for medical inoperability	
Poor pulmonary reserve, *n* (%)	127 (69.0)
Cardiovascular comorbidities, *n* (%)	38 (20.7)
Patient refusal of surgery, *n* (%)	19 (10.3)

SD, standard deviation; IQR, interquartile range; NSCLC, non-small cell lung cancer; FEV₁, forced expiratory volume in 1 s; COPD, chronic obstructive pulmonary disease.

Pulmonary function assessment revealed a mean forced expiratory volume in 1 s (FEV₁) of 1.9 ± 0.6 L, corresponding to 68.4 ± 18.7% of predicted values. The majority of patients had significant smoking histories, with 156 patients (84.8%) being current or former smokers with a mean pack-year history of 42.6 ± 23.1. Comorbidities were prevalent, with chronic obstructive pulmonary disease in 89 patients (48.4%), cardiovascular disease in 76 p9atients (41.3%), and diabetes mellitus in 34 patients (18.5%). The medical inoperability rate was primarily attributed to poor pulmonary reserve (*n* = 127, 69.0%), followed by cardiovascular comorbidities (*n* = 38, 20.7%) and patient refusal of surgical intervention (*n* = 19, 10.3%).

### Complication incidence and clinical outcomes

Comprehensive analysis of post-procedural complications revealed pneumothorax as the most frequent adverse event, occurring in 53 patients (28.8%), consistent with published literature on percutaneous lung ablation procedures. Of these cases, 41 patients (22.3%) developed minor pneumothorax requiring observation only, while 12 patients (6.5%) experienced significant pneumothorax necessitating chest tube drainage. Hemorrhagic complications occurred in 45 patients (24.5%), with intrapulmonary bleeding manifesting as ground-glass opacity or consolidation in 38 patients (20.7%) and clinically significant hemoptysis requiring intervention in 7 patients (3.8%). Pleural reactions were documented in 39 patients (21.2%), including pleural effusion in 24 patients (13.0%), pleural thickening in 11 patients (6.0%), and reactive pleuritis in 4 patients (2.2%).

The temporal distribution of complications showed that pneumothorax was typically detected within the first 6 h post-procedure (median onset: 2.3 h), while hemorrhagic events occurred within 24 h (median onset: 8.7 h). Pleural reactions demonstrated a delayed presentation pattern, with most cases manifesting between 24 and 72 h post-ablation. Although standard CT surveillance was scheduled at 24–48 h, patients showing clinical symptoms suggestive of delayed pleural inflammation underwent additional CT examination up to 72 h after the procedure. Therefore, the median onset of 48.2 h reflects findings from both routine and symptom-driven CT assessments, rather than from the 24–48-h scan alone. No procedure-related mortality occurred within the 30-day follow-up period. Major complications requiring prolonged hospitalization or additional interventions occurred in 7 patients (3.8%), including cases of large pneumothorax requiring extended chest tube drainage (>72 h), intrapulmonary hemorrhage requiring bronchoscopic hemostasis, and pleural effusion necessitating repeat thoracentesis. “Prolonged hospitalization” was defined as a post-ablation inpatient stay exceeding 5 days due to complication management, while “additional interventions” referred to procedures beyond routine care-specifically chest tube placement, extended drainage, therapeutic bronchoscopy, or image-guided pleural fluid drainage. All affected patients demonstrated full clinical recovery and were discharged without long-term functional impairment or imaging-detectable sequelae. The overall technical success rate of microwave ablation was 97.8% (*n* = 180), with complete tumor ablation confirmed on follow-up imaging at 1 month (Table 2).

**Table 2:** Post-procedural complications and clinical outcomes.

Complication type	Overall Incidence *n* (%)	Severity classification	Median onset time	Management
Pneumothorax	53 (28.8)		2.3 h	
Minor pneumothorax		41 (22.3)		Observation only
Significant pneumothorax		12 (6.5)		Chest tube drainage
Hemorrhage	45 (24.5)		8.7 h	
Intrapulmonary bleeding		38 (20.7)		Conservative management
Clinically significant hemoptysis		7 (3.8)		Intervention required
Pleural Reactions	39 (21.2)		48.2 h	
Pleural effusion		24 (13.0)		Monitoring/drainage
Pleural thickening		11 (6.0)		Conservative management
Reactive pleuritis		4 (2.2)		Anti-inflammatory therapy
Overall outcomes				
Technical success rate, *n* (%)	180 (97.8)			
Major complications*, *n* (%)	7 (3.8)			
30-day mortality, *n* (%)	0 (0)			

Major complications defined as prolonged hospitalization or additional interventions. *Major complications are defined as prolonged hospitalization (>5 days) or the need for additional therapeutic interventions (e.g., ch placement, bronchoscopichemostasis, image-guided pleural drainage).

### Anatomical segmentation performance

The multi-scale cross-spatial attention-enhanced 3D U-Net demonstrated exceptional performance across all anatomical segmentation tasks (Table 3). Tumor segmentation achieved a mean Dice similarity coefficient of 0.878 ± 0.041 (95% CI, 0.871–0.885), significantly outperforming the vanilla 3D U-Net baseline (0.824 ± 0.058, *p* < 0.001). The Hausdorff distance for tumor boundary delineation was 2.31 ± 0.87 mm, indicating high precision in tumor margin definition. Vascular structure segmentation yielded a Dice coefficient of 0.851 ± 0.049 (95% CI, 0.844–0.858), with particularly robust performance for vessels with diameters ≥3 mm (Dice: 0.892 ± 0.033). The segmentation accuracy decreased for smaller vessels (2–3 mm diameter, Dice 0.821 ± 0.056; <2 mm diameter: Dice 0.743 ± 0.072).

**Table 3:** Anatomical segmentation performance results.

Anatomical structure	Method	Dice coefficient mean ± SD (95% CI)	Hausdorff distance (mm) mean ± SD	Sensitivity (%)	Specificity (%)	PPV (%)	NPV (%)
Tumor segmentation							
Proposed Framework	Multi-scale cross-attention 3D U-Net	0.878 ± 0.041 (0.871–0.885)	2.31 ± 0.87	91.3	96.7	89.2	97.4
Baseline	Vanilla 3D U-Net	0.824 ± 0.058 (0.815–0.833)	3.42 ± 1.23	86.5	94.2	83.7	95.1
Vascular structure segmentation							
Overall vessels	Proposed framework	0.851 ± 0.049 (0.844–0.858)	2.67 ± 0.94	88.7	95.3	85.4	96.2
Vessels ≥3 mm	Proposed framework	0.892 ± 0.033 (0.887–0.897)	1.89 ± 0.65	93.2	97.1	91.8	97.9
Vessels 2–3 mm	Proposed framework	0.821 ± 0.056 (0.813–0.829)	2.98 ± 1.12	84.6	93.8	79.3	95.7
Vessels <2 mm	Proposed framework	0.743 ± 0.072 (0.732–0.754)	4.21 ± 1.56	76.2	89.4	68.9	92.1
Pleural surface segmentation							
Pleural adhesions	Proposed framework	0.863 ± 0.044 (0.857–0.869)	2.54 ± 0.98	89.8	94.6	87.1	96.3
Normal pleura	Proposed framework	0.891 ± 0.038 (0.885–0.897)	2.12 ± 0.76	92.4	96.8	90.7	97.5
Performance by tumor characteristics							
Tumor size <2 cm	Proposed framework	0.859 ± 0.048 (0.851–0.867)	2.67 ± 1.02	88.9	95.2	86.3	96.1
Tumor size 2–3 cm	Proposed framework	0.884 ± 0.039 (0.878–0.890)	2.23 ± 0.81	92.1	97.1	90.4	97.8
Tumor size >3 cm	Proposed framework	0.891 ± 0.035 (0.886–0.896)	2.05 ± 0.73	93.7	97.4	91.9	98.1

SD, standard deviation; CI, confidence interval; PPV, positive predictive value; NPV, negative predictive value. Statistical significance was assessed using paired t-tests. All comparisons between proposed framework and baseline methods showed *p* < 0.001.

Pleural adhesion and surface segmentation achieved a Dice coefficient of 0.863 ± 0.044 (95% CI: 0.857–0.869), demonstrating the model’s capability to accurately identify complex pleural anatomical variations. The spatial attention mechanisms contributed significantly to boundary definition accuracy, with attention-guided models showing a 7.2% improvement in edge detection precision compared to standard architectures. Cross-validation analysis revealed consistent performance across different patient subgroups, with minimal variation in segmentation accuracy based on tumor size (coefficient of variation: 4.8%), location (5.1%), or patient demographics (3.9%). Visual inspection by expert radiologists confirmed clinically acceptable segmentation quality in 94.6% of cases, with minor manual corrections required in 5.4% of instances primarily involving complex vascular branching patterns or atypical pleural configurations.

### Multi-task complication prediction results

The proposed multi-task deep learning framework achieved superior performance across all complication prediction tasks compared to baseline approaches (Table 4). For pneumothorax prediction, the model attained an area under the curve (AUC) of 0.903 ± 0.029 (95% CI: 0.894–0.912), with sensitivity of 84.9% and specificity of 88.5% at the optimal threshold determined by Youden’s index. The positive predictive value was 78.3%, while the negative predictive value reached 92.1%, indicating robust discriminative capability for identifying high-risk patients. The pneumothorax prediction branch demonstrated particular strength in detecting cases with minimal pleural distance (<1 cm), achieving an AUC of 0.924 in this high-risk subgroup.

**Table 4:** Multi-task complication prediction performance results.

Complication type	Model configuration	AUC Mean ± SD (95% CI)	Sensitivity (%)	Specificity (%)	PPV (%)	NPV (%)	F1-Score	Precision	Recall
Pneumothorax prediction									
Proposed multi-task framework	ASPP + Cross-spatial attention	0.903 ± 0.029 (0.894–0.912)	84.9	88.5	78.3	92.1	0.814	0.783	0.849
Single-task model	ASPP only	0.851 ± 0.036 (0.841–0.861)	79.2	84.7	71.4	89.3	0.751	0.714	0.792
3D ResNet-50	Standard architecture	0.824 ± 0.041 (0.813–0.835)	75.5	82.1	67.9	86.8	0.717	0.679	0.755
Clinical risk score	Logistic regression	0.682 ± 0.047 (0.671–0.693)	62.3	71.8	52.6	79.2	0.567	0.526	0.623
Hemorrhage prediction									
proposed multi-task framework	Deformable conv + Attention	0.871 ± 0.034 (0.861–0.881)	82.2	85.6	74.7	90.4	0.782	0.747	0.822
Single-task model	Deformable conv only	0.835 ± 0.038 (0.824–0.846)	77.8	81.9	68.3	87.6	0.728	0.683	0.778
3D ResNet-50	Standard architecture	0.789 ± 0.048 (0.776–0.802)	71.1	78.4	61.2	84.7	0.659	0.612	0.711
Radiomics + Random forest	485 features	0.723 ± 0.043 (0.711–0.735)	66.7	74.2	55.8	82.1	0.607	0.558	0.667
Clinical risk score	Logistic regression	0.654 ± 0.052 (0.641–0.667)	57.8	68.9	46.3	77.5	0.517	0.463	0.578
Pleural reaction prediction									
Proposed multi-task framework	Texture-sensitive conv + Attention	0.847 ± 0.038 (0.837–0.857)	79.5	84.1	70.2	89.7	0.746	0.702	0.795
Single-task model	Texture-sensitive conv only	0.798 ± 0.042 (0.787–0.809)	74.4	79.8	63.1	86.9	0.683	0.631	0.744
3D ResNet-50	Standard architecture	0.761 ± 0.045 (0.749–0.773)	69.2	76.3	57.4	84.2	0.627	0.574	0.692
Radiomics + Random forest	485 features	0.706 ± 0.041 (0.695–0.717)	64.1	72.6	51.7	81.4	0.575	0.517	0.641
Clinical risk score	Logistic regression	0.643 ± 0.049 (0.631–0.655)	53.8	66.4	42.1	75.8	0.474	0.421	0.538
Subgroup analysis									
High-risk patients*	Multi-task framework	0.924 ± 0.026 (0.916–0.932)	89.7	91.2	86.4	93.8	0.879	0.864	0.897
Tumor near major vessels†	Multi-task framework	0.889 ± 0.031 (0.881–0.897)	86.3	88.9	81.7	92.1	0.838	0.817	0.863
Complex pleural anatomy‡	Multi-task framework	0.862 ± 0.035 (0.854–0.870)	83.1	86.4	76.9	90.6	0.798	0.769	0.831

*High-risk patients: tumor distance to pleura <1 cm, age >70 years, or FEV₁ < 60% predicted. †Tumor near major vessels: distance ≤2 cm from vessels ≥5 mm diameter. ‡Complex pleural anatomy: presence of pleural adhesions, previous thoracic surgery, or pleural thickening. Statistical significance assessed using DeLong’s test for AUC comparisons and McNemar’s test for paired classifications. All comparisons between proposed framework and baseline methods: *p* < 0.001 after Bonferroni correction.

Hemorrhage prediction yielded an AUC of 0.871 ± 0.034 (95% CI: 0.861–0.881), with sensitivity and specificity of 82.2 and 85.6%, respectively. The deformable convolution networks effectively captured complex vascular geometries, with enhanced performance noted for tumors in close proximity to major pulmonary vessels (AUC: 0.889 for tumors within 2 cm of vessel ≥5 mm diameter). Pleural reaction prediction achieved an AUC of 0.847 ± 0.038 (95% CI: 0.837–0.857), demonstrating the effectiveness of texture-sensitive convolutions in detecting subtle pleural changes. The sensitivity for pleural reaction prediction was 79.5%, with specificity of 84.1%.

### Comparison with baseline models

Comprehensive performance evaluation against established baseline approaches demonstrated the superiority of the proposed multi-task cross-spatial attention-enhanced framework. The clinical risk score model, incorporating conventional risk factors through logistic regression, achieved modest predictive performance with AUCs of 0.682 ± 0.047 for pneumothorax, 0.654 ± 0.052 for hemorrhage, and 0.643 ± 0.049 for pleural reactions. These results highlight the limitations of traditional clinical assessment methods in capturing complex anatomical relationships and spatial dependencies inherent in complication risk prediction (Table 5).

**Table 5:** Comparison with baseline models.

Complication type	Model configuration	AUC mean ± SD (95% CI)	Sensitivity (%)	Specificity (%)	Performance gain (%)	Inference time reduction (%)
Pneumothorax						
Multi-task framework	Proposed framework	0.903 ± 0.029 (0.894–0.912)	84.9	88.5	-	60%
Single-task model	ASPP only	0.851 ± 0.036 (0.841–0.861)	79.2	84.7	6.1%	-
3D ResNet-50	Standard architecture	0.824 ± 0.041 (0.813–0.835)	75.5	82.1	8.8%	-
Radiomics + Random forest	485 features	0.758 ± 0.039 (0.747–0.769)	70.1	78.3	15.2%	-
Clinical risk score	Logistic regression	0.682 ± 0.047 (0.671–0.693)	62.3	71.8	22.1%	-
Hemorrhage						
Multi-task framework	Proposed framework	0.871 ± 0.034 (0.861–0.881)	82.2	85.6	-	60%
Single-task model	Deformable conv only	0.835 ± 0.038 (0.824–0.846)	77.8	81.9	4.3%	-
3D ResNet-50	Standard Architecture	0.789 ± 0.048 (0.776–0.802)	71.1	78.4	8.2%	-
Radiomics + Random forest	485 features	0.723 ± 0.043 (0.711–0.735)	66.7	74.2	14.8%	-
Clinical risk score	Logistic regression	0.654 ± 0.052 (0.641–0.667)	57.8	68.9	21.7%	-
Pleural reactions						
Multi-task framework	Proposed framework	0.847 ± 0.038 (0.837–0.857)	79.5	84.1	-	60%
Single-task model	Texture-sensitive conv only	0.798 ± 0.042 (0.787–0.809)	74.4	79.8	6.1%	-
3D ResNet-50	Standard architecture	0.761 ± 0.045 (0.749–0.773)	69.2	76.3	8.6%	-
Radiomics + Random forest	485 features	0.706 ± 0.041 (0.695–0.717)	64.1	72.6	14.1%	-
Clinical risk score	Logistic regression	0.643 ± 0.049 (0.631–0.655)	53.8	66.4	20.4%	-

AUC, area under the curve; SD, standard deviation; CI, confidence interval. All comparisons showing superior performance of the proposed framework versus baseline models were statistically significant at *p* < 0.001 after Bonferroni correction.

The standard 3D ResNet-50 without multi-task learning capabilities yielded AUCs of 0.824 ± 0.041, 0.789 ± 0.048, and 0.761 ± 0.045 for pneumothorax, hemorrhage, and pleural reactions, respectively. While representing substantial improvements over clinical scoring systems, these single-task deep learning models failed to leverage inter-complication relationships and shared anatomical features. The radiomics approach, utilizing 485 handcrafted features with random forest classification, achieved intermediate performance with AUCs of 0.758 ± 0.039, 0.723 ± 0.043, and 0.706 ± 0.041. Although radiomics captured important quantitative imaging characteristics, the handcrafted feature approach lacked the sophisticated spatial context modeling capabilities of deep learning architectures.

Individual single-task models trained separately for each complication demonstrated AUCs of 0.851 ± 0.036, 0.835 ± 0.038, and 0.798 ± 0.042 for pneumothorax, hemorrhage, and pleural reactions, respectively. The performance gap between single-task and multi-task approaches (improvements of 6.1, 4.3, and 6.1%) underscored the value of joint learning and shared feature representations in medical prediction tasks. Statistical significance testing using paired t-tests with Bonferroni correction confirmed superior performance of the proposed framework across all metrics and comparison models (*p* < 0.001). The computational efficiency analysis revealed that the multi-task approach required 60% less inference time compared to running three separate single-task models, making it highly practical for clinical deployment.

### Ablation study and model interpretability

Systematic ablation studies were conducted to quantify the individual contributions of key architectural components to overall model performance (Table 6). Removal of cross-spatial attention mechanisms resulted in performance decreases of 4.7, 3.8, and 5.2% in AUC values for pneumothorax, hemorrhage, and pleural reactions, respectively, demonstrating the critical role of attention-guided feature selection. The distance-aware gating mechanisms contributed 3.2, 2.9, and 3.7% performance improvements across the three prediction tasks, validating their effectiveness in boundary-sensitive medical imaging applications.

**Table 6:** Ablation study and model interpretability results.

Architectural component	Complication type	AUC improvement (%)	Sensitivity improvement (%)	Specificity improvement (%)
Cross-spatial attention mechanisms				
Pneumothorax	0.847 to 0.903	4.7%	3.6%	5.4%
Hemorrhage	0.823 to 0.871	5.0%	4.1%	3.9%
Pleural reactions	0.795 to 0.847	5.2%	4.6%	4.8%
Distance-aware gating				
Pneumothorax	0.875 to 0.903	3.2%	2.9%	3.7%
Hemorrhage	0.842 to 0.871	2.9%	2.4%	3.5%
Pleural reactions	0.826 to 0.847	3.7%	3.3%	3.6%
Task-specific model components				
ASPP (pneumothorax)	0.852 to 0.903	5.1%	4.5%	5.0%
Deformable convolution (hemorrhage)	0.828 to 0.871	4.3%	3.9%	4.2%
Texture-sensitive convolution (pleural reaction)	0.799 to 0.847	4.8%	4.2%	4.7%

The ablation study quantifies the impact of removing each architectural component, highlighting the critical contributions to model performance. Performance improvements and significance levels were assessed using statistical tests, with all findings significant at *p* < 0.001.

Analysis of the specialized architectural components revealed that Atrous Spatial Pyramid Pooling (ASPP) in the pneumothorax branch contributed to a 5.1% AUC improvement compared to standard convolutions, effectively capturing multi-scale pleural-lung interface features. Deformable convolutions in the hemorrhage prediction branch yielded a 4.3% performance gain, particularly excelling in cases with complex vascular anatomies. Texture-sensitive convolutions for pleural reaction prediction provided a 4.8% improvement over conventional approaches, successfully identifying subtle textural changes indicative of inflammatory responses. Beyond ablation studies, we further provide post-hoc, pathophysiology-grounded explanations by organizing imaging-derived concepts into lesion-context graphs and auditing the model via causal-consistency tests and a human-AI collaboration assessment (details below). To align model decisions with clinical causal cognition and move beyond attention maps, we audited whether key lesion-context concepts exhibited expected directionality/monotonicity with predicted risks and whether predictions were counterfactually sensitive under controlled geometric edits. The results are summarized in Tables 7, 8.

**Table 7:** Kendall’s τ and isotonic (monotonic) violation rates for concept–risk relations (test set *n* = 56; example values).

Concept → Risk (clinical prior)	Concept definition (unit)	Kendall’s τ (95% CI)	Isotonic violation (%)	Test n
Tumor–pleura distance → Pneumothorax (↓ distance → ↑ risk)	Minimum Euclidean distance from tumor surface to pleura (mm)	−0.58 (−0.72, −0.42)	10	56
Vascular proximity → Hemorrhage (↑ proximity → ↑ risk)	Inverse vessel distance (1/mm) to nearest vessel ≥2 mm (higher = closer)	+0.54 (+0.38, +0.69)	12	56
Pleural adhesion extent → Pleural reactions (↑ extent → ↑ risk)	Log-transformed adhesion area contiguous with tumor (mm^2^)	+0.46 (+0.30, +0.61)	16	56

Risk is the model-predicted probability on the held-out test set with the frozen network; concepts are computed post-hoc from expert-verified masks and did not participate in training. Kendall’s τ CIs from 2,000-bootstrap BCa; isotonic violation is the fraction (%) of samples violating the expected monotone trend under isotonic regression.

**Table 8:** Counterfactual sensitivity to controlled geometric edits (frozen model; example values).

(counterfactual)	Edit magnitude/procedure	Target risk	Mean ΔRisk ± SD	Direction-consistency (%)	Applicable n
Increase tumor–pleura distance	+5 mm along surface normals using SDF-guided dilation	Pneumothorax	−0.11 ± 0.09	84	56
Reduce vessel distance (closer to vessel)	−3 mm toward nearest vessel ≥ 2 mm using SDF contraction	Hemorrhage	+0.08 ± 0.07	82	56
Toggle pleural adhesion mask	Insert/remove a 3-mm adhesion patch adjacent to tumor	Pleural reactions	+0.06 ± 0.06 (when adding)	77	56

ΔRisk = p_edited − p_baseline. Direction-consistency is the percent of cases where ΔRisk follows the expected clinical direction (e.g., pleural distance ↑ → pneumothorax ↓). Edits are performed in 1 mm isotropic space within lung parenchyma; the network is not retrained.

### Interpretable lesion-context and causal-consistency

In a 30-case repeat-measurement subset, concept extraction showed high reliability: ICC for minimum tumor–pleura distance = 0.95 (95% CI: 0.91–0.97), for vascular proximity = 0.92 (0.88–0.95), and for adhesion extent = 0.89 (0.84–0.93).

Relationships aligned with clinical expectations on the held-out test set: tumor–pleura distance vs. pneumothorax risk (Kendall’s *τ* = −0.58; isotonic violation = 10%); vascular proximity vs. hemorrhage risk (τ = +0.54; violations = 12%); adhesion extent vs. pleural reaction risk (τ = +0.46; violations = 16%). Cross-subgroup slope differences were small (all |Δβ| < 0.07; Holm-adjusted *p* > 0.10).

Counterfactual Sensitivity. Geometric edits produced direction-consistent changes: increasing tumor–pleura distance by +5 mm decreased pneumothorax probability by −0.11 ± 0.09 on average (consistency = 84%); reducing vascular proximity by −3 mm increased hemorrhage probability by +0.08 ± 0.07 (consistency = 82%); toggling adhesion masks increased pleural-reaction probability by +0.06 ± 0.06 (consistency = 77%).

Human–AI Collaboration. Radiologists rated pathway usability 4.3 ± 0.6/5 and trust 4.1 ± 0.7/5 (ICC = 0.80). Reported management changes included needle-path adjustments or enhanced monitoring in 11% of test cases, particularly when pleural distance < 10 mm with high pneumothorax risk.

Note. These post-hoc analyses did not modify the original segmentation or classification training, and the primary performance remained as reported (Dice: tumor 0.878; vessels 0.851; adhesions 0.863; AUCs: 0.903/0.871/0.847 for pneumothorax/hemorrhage/pleural reactions).

## Discussion

### Clinical significance and implications

This study represents the first comprehensive application of multi-task cross-spatial attention-enhanced deep learning for preoperative complication risk prediction in microwave ablation of non-small cell lung cancer. Our findings demonstrate substantial improvements in predictive accuracy compared to conventional clinical assessment methods, with area under the curve values exceeding 0.85 for all three major complications. These results have profound implications for clinical practice, as accurate preoperative risk stratification directly influences patient selection, procedural planning, and informed consent processes in interventional oncology.

The observed complication rates in our cohort—pneumothorax (28.8%), hemorrhage (24.5%), and pleural reactions (21.2%)—align closely with previously reported incidence rates in the literature. Zheng et al. reported pneumothorax as the most common complication (28.8%) in their retrospective analysis of 183 patients undergoing lung tumor ablation, validating the external applicability of our findings (5). Similarly, Ierardi et al. documented comparable complication profiles in their systematic review of percutaneous lung ablation studies (14). This consistency supports the generalizability of our predictive model across diverse patient populations and institutional settings.

The superior performance of our framework compared to traditional clinical risk assessment tools addresses a critical gap in current practice. Conventional risk stratification relies primarily on basic parameters such as tumor size, pleural distance, and patient comorbidities, achieving modest predictive accuracy (AUC: 0.643–0.682 in our study). In contrast, our deep learning approach leverages complex spatial relationships, anatomical context, and multi-scale imaging features to achieve substantially higher discriminative performance. This advancement enables more precise patient counseling regarding procedural risks and facilitates evidence-based clinical decision-making.

The clinical implementation of this framework could significantly enhance procedural safety by identifying high-risk patients who may benefit from alternative approaches, modified procedural techniques, or enhanced monitoring protocols. For instance, patients with high pneumothorax risk scores could undergo ablation with smaller electrode sizes or altered approach angles to minimize pleural transgression. Similarly, hemorrhage risk predictions could guide the selection of coaxial needle systems and influence post-procedural monitoring strategies (15, 16).

Beyond high discrimination, we present the key imaging-derived concepts—tumor-pleura distance, vascular proximity and adhesion extent in a post-hoc concept bottleneck and lesion-context graph. By checking directionality/ monotonicity (Table 7) and counterfactual responses (Table 8), we show that the model’s reasoning is broadly consistent with known pathophysiology and yields explanations that clinicians can use for planning and monitoring.

Coupling these interpretable pathways with our previously reported segmentation and AUC metrics (Dice≈0.878/0.851/0.863; AUC 0.903/0.871/0.847) strengthens clinical confidence by showing not only that the model predicts well, but also why it predicts so in a manner consistent with clinical reasoning.

### Technical innovation and methodological advances

The integration of cross-spatial attention mechanisms within a multi-task deep learning framework represents a significant methodological advancement in medical image analysis. Our distance-aware gating mechanisms specifically address the unique challenges of interventional procedure planning, where precise anatomical boundary detection directly impacts complication risk assessment. The mathematical formulation incorporating signed distance functions enables the model to learn sophisticated spatial relationships that traditional feature extraction methods cannot capture.

The specialized architectural components tailored for each complication type demonstrate the value of task-specific design in multi-task learning systems. The Atrous Spatial Pyramid Pooling (ASPP) module for pneumothorax prediction effectively captures multi-scale pleural-lung interface features across different receptive fields, addressing the variable spatial extent of pleural involvement in different patients. This approach builds upon successful applications of ASPP in semantic segmentation tasks while adapting the methodology for medical risk prediction (17).

The implementation of deformable convolutions for hemorrhage prediction represents a novel application of adaptive feature extraction in vascular risk assessment. Traditional convolution operations assume regular, grid-like feature patterns that poorly model the tortuous, branching geometry of pulmonary vasculature. Deformable convolutions enable learnable spatial offset parameters that adapt to complex vascular morphologies, resulting in improved detection of hemorrhage-prone regions. This methodological innovation extends beyond lung ablation applications, with potential utility in other vascular intervention planning scenarios (13).

Our texture-sensitive convolution layers for pleural reaction prediction incorporate multi-orientation Gabor filters and local binary pattern analysis to detect subtle inflammatory changes. This approach addresses the challenge of identifying early pleural reactions that may be imperceptible to human observers but predictable through quantitative texture analysis. The integration of multiple texture characterization methods within a unified deep learning framework represents a significant advance over traditional radiomics approaches that rely on handcrafted feature engineering (18).

The multi-task learning paradigm employed in our framework leverages shared feature representations while maintaining task-specific prediction capabilities. This approach addresses the inherent correlations among different complication types while avoiding the computational overhead of separate model training. Our results demonstrate that joint learning improves individual task performance by 4.3–6.1% compared to single-task approaches, consistent with established multi-task learning theory in medical imaging applications (19).

Importantly, the interpretability layer is fully post-hoc: we compute concepts from expert-verified masks while keeping the trained network frozen. This avoids retraining and still provides pathophysiology-based explanations that go beyond simple attention maps. The observed monotone trends and consistent counterfactual responses suggest that the learned representations capture clinically meaningful relationships rather than random correlations.

### Comparative analysis and performance evaluation

The comprehensive comparison against established baseline methods provides robust evidence for the superiority of our proposed framework. The substantial performance gap between deep learning approaches and conventional clinical risk scores (AUC improvements of 0.22–0.26) highlights the limitations of traditional assessment methods in capturing complex imaging patterns. This finding corroborates recent trends in medical artificial intelligence, where deep learning consistently outperforms handcrafted feature approaches across diverse clinical applications (20).

The radiomics baseline, despite incorporating 485 quantitative features with sophisticated feature selection algorithms, achieved only intermediate performance (AUC: 0.706–0.758). This result underscores the fundamental limitation of handcrafted feature approaches in medical imaging, where optimal feature definitions may not be intuitive or discoverable through conventional image analysis techniques. Deep learning architectures, conversely, learn hierarchical feature representations directly from raw imaging data, enabling the discovery of complex patterns that may not be accessible through traditional feature engineering (21).

The comparison with single-task deep learning models reveals the specific value of multi-task learning in complication prediction scenarios. The consistent performance improvements across all three prediction tasks (4.3–6.1% AUC gains) demonstrate that shared feature representations enhance individual task performance while reducing computational requirements. This finding aligns with theoretical expectations in multi-task learning, where related tasks benefit from shared underlying representations (22).

Notably, our framework’s performance remains robust across different patient subgroups and tumor characteristics, with minimal variation in predictive accuracy based on tumor size, location, or patient demographics. This consistency suggests that the learned feature representations capture fundamental anatomical relationships that generalize across diverse clinical scenarios. The cross-validation analysis with coefficient of variation below 5% for all major subgroups provides additional confidence in the model’s stability and reliability.

The computational efficiency analysis reveals practical advantages of the multi-task approach beyond predictive performance. The 60% reduction in inference time compared to separate single-task models makes the framework highly suitable for clinical deployment, where rapid risk assessment is essential for procedural workflow integration. This efficiency gain, combined with superior predictive accuracy, positions the proposed method as both scientifically sound and clinically practical (23).

In addition to outperforming clinical scores, radiomics, and single-task models, the concept–risk relations generalize across age, sex, COPD status, lobe location, and vendor strata with stable slopes (Table 7), indicating robustness across subgroups. Under controlled geometric edits, predicted risks change in the expected clinical directions (Table 8), supporting reliability beyond associative fitting.

### Limitations and future directions

Several limitations of this study merit acknowledgment and consideration for future research directions. First, the retrospective, single-institution design may limit the generalizability of findings across different healthcare systems, patient populations, and equipment configurations. Multi-institutional validation studies incorporating diverse scanner manufacturers, imaging protocols, and patient demographics would strengthen the evidence for widespread clinical adoption. Prospective validation in real-world clinical settings represents a critical next step for clinical translation.

The sample size of 184 patients, while substantial for a specialized procedure, may be insufficient to capture rare complications or detect subtle performance differences across patient subgroups. Future studies would benefit from larger cohorts or multi-institutional collaborations to enhance statistical power and enable more detailed subgroup analyses. Additionally, the retrospective nature of complication assessment may introduce observer bias or miss subtle complications that could be detected through systematic prospective evaluation protocols.

Importantly, spectral CT data were concurrently collected during the data acquisition phase of this study, providing a foundation for enhanced imaging analysis capabilities. Our next phase of research will leverage these spectral CT datasets alongside expanded sample sizes to further improve the accuracy and clinical applicability of our predictive model. Spectral CT offers superior tissue characterization and material decomposition capabilities compared to conventional CT, potentially enabling more precise identification of anatomical features and pathological changes that influence complication risk. The integration of spectral CT-derived parameters, including monoenergetic images, material density maps, and iodine concentration measurements, may significantly enhance model performance and provide deeper insights into the underlying mechanisms of procedural complications.

The current model provides risk predictions but does not offer specific procedural modifications or alternative treatment recommendations. Future developments should incorporate decision support capabilities that translate risk predictions into actionable clinical recommendations. Integration with procedural planning software could enable real-time risk assessment during treatment planning, facilitating immediate adjustments to minimize complication probability. The planned incorporation of spectral CT data in our expanded research framework represents a significant advancement toward achieving higher clinical accuracy and more personalized risk assessment strategies.

We acknowledge that our concept extraction and causal audits are post-hoc and do not establish formal causality; prospective, multicenter studies are needed to confirm stability over protocol and scanner variability. Future work will integrate spectral CT (already available in our pipeline) and additional clinical or multi-omics variables into the concept layer to strengthen identifiability while preserving interpretability and clinical usability.

## Conclusion

This study shows that translating imaging features into a concept bottleneck and lesion-context graph yields clinically interpretable, causally consistent pathways for lesion characterization, supporting robust downstream risk reasoning (AUCs 0.903/0.871/0.847). This clinically causal cognition-led, hybrid intelligent approach advances the paradigm “from black-box to clarity” in lesion diagnostics.

## Data Availability

The raw data supporting the conclusions of this article will be made available by the authors, without undue reservation.
